# Frequency and impact of enteric hyperoxaluria in pediatric short bowel syndrome: a retrospective single centre study

**DOI:** 10.3389/fped.2023.1157696

**Published:** 2023-07-12

**Authors:** Jan Thomas Schaefer, Susanne Schulz-Heise, Aline Rueckel, Manfred Rauh, Joerg Juengert, Matthias Galiano, Norbert Meier, Joachim Woelfle, Mario Schiffer, André Hoerning

**Affiliations:** ^1^ Department of Paediatrics and Adolescent Medicine, University Hospital Erlangen, Friedrich-Alexander-University (FAU) Erlangen-Nürnberg, Erlangen, Germany; ^2^ Research Center on Rare Kidney Diseases (RECORD), University Hospital Erlangen, Erlangen, Germany; ^3^ Institute of Radiology, University Hospital Erlangen, Friedrich-Alexander-Universität (FAU) Erlangen-Nürnberg, Erlangen, Germany; ^4^ Department of Nephrology and Hypertension, University Hospital Erlangen, Friedrich-Alexander-Universität (FAU) Erlangen-Nürnberg, Erlangen, Germany

**Keywords:** hyperoxaluria, enteric hyperoxaluria, short bowel syndrome, nephrolithiasis, kidney stone, pediatric, intestinal failure

## Abstract

**Objectives:**

The survival of pediatric patients with short bowel syndrome has improved in recent years. Enteric hyperoxaluria as a pathophysiological consequence has been hardly addressed so far. It can be associated with nephrolithiasis, nephrocalcinosis or even renal insufficiency. We assessed the prevalence of hyperoxaluria and its pathogenic consequences in a retrospective single centre study over the last 12 years.

**Methods:**

We conducted an internal database search for all pediatric patients suffering from short bowel syndrome treated from 2010 to 2022 in the department of pediatric gastroenterology as well as the pediatric nephrology and dialysis unit. Out of 56 patients identified, 26 patients were analysed for etiology of short bowel syndrome, renal excretion of oxalate (24/26), remaining short bowel and large intestinal length as well as further clinical parameters such as eGFR, nephrocalcinosis/urinary stone formation or stool frequency.

**Results:**

Hyperoxaluria was detected in 14/26 patients (54%). Nephrocalcinosis was present in four patients. Out of these four patients, hyperoxaluria could be proven (21% of all hyperoxaluric patients) in three cases, one hyperoxaluric patient had nephrolithiasis (7%). In one patient hyperoxaluria lead to end stage renal disease. We found that 80% of patients with volvulus developed enteric hyperoxaluria. None of the investigated factors had an effect on oxalate excretion.

**Conclusion:**

Enteric hyperoxaluria is a relevant pathophysiological finding in patients with short bowel syndrome occurring in about 50% of our cohort with multiple pathogenic complications. Regular screening for hyperoxaluria may be implemented in medical care for patients with short bowel syndrome. If necessary, prophylaxis, e.g., dietary advice or metaphylaxis should be initiated.

## Introduction

In recent years, medical advances have resulted in an improvement of treatment quality and subsequently longer survival rate of pediatric patients with short bowel syndrome (SBS) or chronic intestinal failure (1). However, the higher survival rate is accompanied by an intensive need to care for such patients, some of whom are very complex and dependent on lifelong close and strict medical supervision. In addition to central line associated bloodstream infections, recurrent catheter-related thrombosis, intestinal failure-associated liver disease, an overall impact on quality of life, metabolic bone problems and malnutrition, other consequences demand awareness and treatment.

Enteric hyperoxaluria as a pathophysiological consequence in pediatric patients with short bowel syndrome has been addressed in only some case reports and few single center studies (2–5). It can lead to nephrolithiasis or nephrocalcinosis, even rarely to terminal renal failure (6, 7). Regarding development of nephrolithiasis, it is worthful to mention that hyperoxaluria seems to have a higher impact than hypercalciuria (8). Enteric hyperoxaluria came to be recognised following the widespread introduction of bariatric surgery in the last decades of the previous century (9–11). In addition to patients after bariatric surgery, enteric hyperoxaluria is also found in patients with Crohn's disease or cystic fibrosis, as well as in patients with SBS (2, 3, 12–14). Recently published studies in adult patients with SBS have reported a significantly increased prevalence of nephrolithiasis of approximately 18%–24% compared to the general population (15, 16). In particular, the risk was increased when oral nutrition was given in addition to parenteral nutrition. Studies by Kosar and Roberts showed a high prevalence of hyperoxaluria and altered urinary tract findings beginning with an increase in echogenicity of the kidneys or even nephrocalcinosis in pediatric patients with intestinal failure preponderantly caused by anatomical short bowel syndrome (3, 17). However, they could not find a correlation to the type and composition of nutrition. A correlation between hyperoxaluria and other factors like remaining short bowel length, eGFR and hyperoxaluria could not be seen. Since only few pediatric data for patients with anatomical short bowel syndrome are available, we investigated the incidence of hyperoxaluria in short bowel patients leading to complications such as nephrocalcinosis or nephrolithiasis. The aim further was to determine a possible correlation of hyperoxaluria with risk factors such as the cause for SBS, residual small bowel length, the type of nutrition and the development of nephrocalcinosis or nephrolithiasis.

## Material and methods

### Data assessment

#### Patient population and study design

We conducted an internal database analysis of patients with SBS or extensive bowel resection <18 years of age on admission who were seen in our departments of pediatric gastroenterology as well as the pediatric nephrology and dialysis unit from 2010 to 2022. Data were collected from electronic medical records at the end of 2022.

The definition of Muto et al. was used to define the group of pediatric patients suffering from SBS (18). According to the author, the definition of short bowel syndrome in childhood is met when either permanent (usually 2 months) parenteral nutrition is required or when there is a residual short bowel length of 25%–30% of the age-appropriate expected short bowel length according to Struijs et al. (19). If less than 10–25 cm or <10% of the expected bowel length remains, the term ultra-short bowel syndrome is used. A history of prolonged need (>2 months) for parenteral nutrition was required for being eligible for the study but persistent parenteral nutrition was not a prerequisite at the time of data collection. As hyperoxaluria in SBS patients might play an important role in developing nephrolithiasis, we defined two groups: patients with hyperoxaluria and patients without hyperoxaluria. If no urine production was present, a proven oxalate nephropathy by a specialized nephropathologist in combination with excess level of plasma oxalate was required to establish diagnosis of enteric hyperoxaluria.

#### Study variables

**Figure 1:**
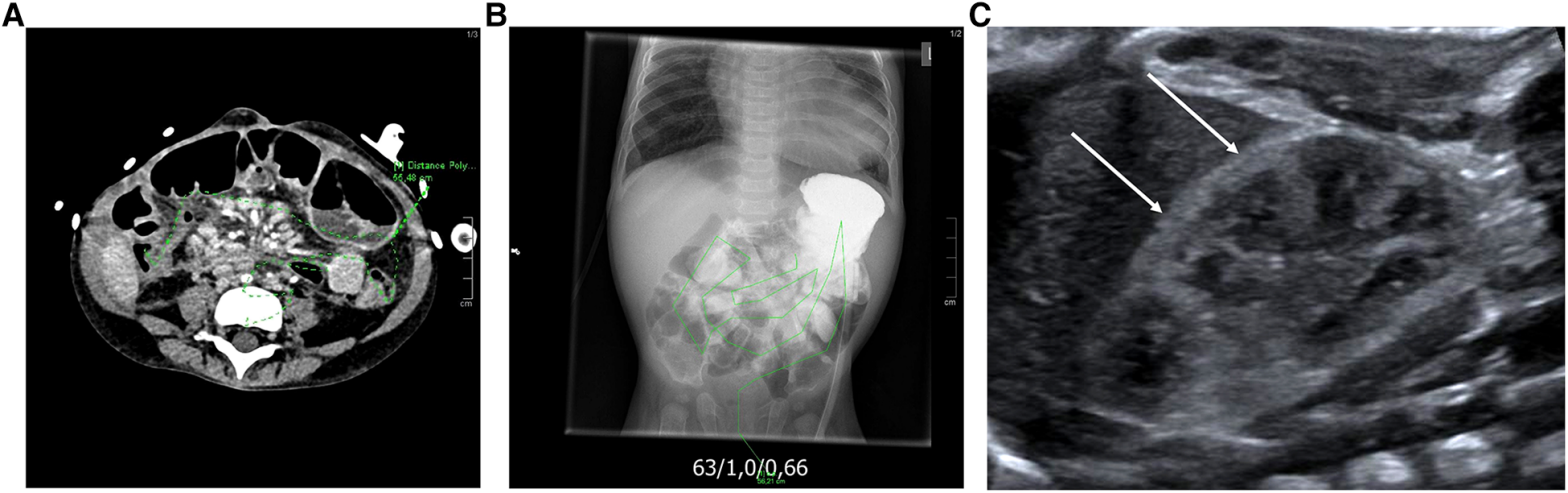
Representative determination of small bowel length in abdominal CT (**A**) and conventional x-ray (**B**) as well as ultrasonographic imaging (**C**) showing hyperechoic cortical halo (arrow) of the right kidney in patient 1 at the age of ca. 3 months, compatible with a beginning cortical nephrocalcinosis.

Demographic data included age, gender, BMI and etiology of SBS. Residual short bowel length was recorded from surgical protocols and from a pediatric radiologist using the most accurate radiological imaging (abdominal CT/MRI if available, otherwise gastrointestinal passage via barium contrast) for bowel length measurement in a blinded fashion as illustrated in Figures 1A,B. Expected small bowel length (SBL) according to Struijs was calculated by the following formula: ln(SBL) = 6.741–80,409/height (cm) (19). This formula was evaluated for premature children of postconceptual age of 24 weeks up to 5 years. To compare results, remaining intestinal length was expressed as percentage of expected length (0%–100%). Large intestinal length was recoded in absolute length, as no relative data comparable to small bowel length according to Struijs is available. Furthermore, it was recorded whether or not there was continuity of the bowel at the time of sample collection. Stool frequency was assessed by medical reports. Patients with small bowel stoma were excluded from stool frequency assessment as these patients have permanent emptying of stool, which makes it impossible to specify a frequency.

Information about the components of the nutrition were taken from the diet plan closest to the sample taken. The presence of nephrocalcinosis or nephrolithiasis (symptomatic or not) was recorded by a patient interview and according to the records (medical report, imaging diagnostics such as sonography of the abdomen, x-ray examinations if available).

The most recent laboratory results of creatinine and cystatin C in the blood, spontaneous urine for creatinine, calcium, oxalate and citrate were analysed. When dietary treatment for enteric hyperoxaluria was performed, oxalate measurements before the start of dietary therapy were taken into account.

All laboratory results were performed in our in-house accredited laboratory (20–24). A cystatin C based eGFR was calculated according to a formula by Grubb (25). Patients on long-term parenteral nutrition are likely to develop an altered body composition with an overall reduced muscle mass, so serum creatinine loses reliability in estimating GFR in this patient group (26–28). Therefore, Grubb's formula was used as it does not depend on body muscle mass like Schwarz formula does. Serum creatinine and cystatin C before dialysis was used for patients with dialysis treatment.

#### Measurement of urinary oxalate

Urine was collected when patients were in our clinic. If measurement was not made immediately, urine was stored at −20°C. Before measurement, specimen temperature was warmed up to 37°C. Measurement of urinary oxalate was conducted according to manufacturer's protocol (enzymatic testing, Trinity Biotech; Bray, Co Wicklow, Ireland) by a photometric analyser (Respons 910, DiaSys Diagnostic Systms GmbH, Holzheim, Germany). Oxalate excretion was expressed in mmol/mol urinary creatinine, as costumary.

### Statistical analysis

Statistical analysis was performed using Graphpad Prism version 9.3.1. Numerical data were expressed as medians with interquartile range (additionally as means with standard deviation in tables). Metric data were analysed using the Mann-Whitney *U* test, as no normal distribution could be assumed after reviewing the frequency histograms. Category variables were presented as frequencies and statistical tests were performed using Fisher's exact test. An alpha value of *p* < 0.05 was considered statistically significant.

For comparison purposes, two age matched control groups (group one: creatinine and oxalate; group 2: cystatin C) were generated from an internal patient database (see Table 1). These were patients with isolated benign familial microhematuria (convenient sampling) who were evaluated for routine clinical care in our pediatric-nephrological outpatient clinic. In these patients, serum creatinine and cystatin C as well as fresh voided spot urine samples for oxalate excretion were measured in our laboratory in the same manner as in SBS patients mentioned above.

**Table 1:** Patient characteristics.

Parameter	Group	Number of values	Minimum	25% percentile	median	75% percentile	Maximum	Range	Mean	Std. deviation	Std. error of mean	*p* value
Gender	Female HOx	9										0.0618
	Male HOx	5										
	Female noHOx	3										
	Male noHOx	9										
Age (months)	HOx	13	15	32.75	48	159.5	279	264	89.71	80.43	21.5	0.9396
	noHOx	12	7	30.75	70.5	123.8	186	179	80.75	59.82	17.27	
	Ctrl Cr/Ox	50	0	53.75	67	99.5	195	195	77.36	35.68	5.05	to HOx: 0.3118 to noHOx: 0.9195
	Ctrl eGFR	28	42	54	77.5	85.75	141	99	74.39	65.38	24.16	to HOx: 0.305 to noHOx: 0.8323
BMI (kg/m^2^)	HOx	14	11.96	14.68	15.85	18.66	22.9	10.94	16.56	2.8	0.75	0.6946
	noHOx	12	13.8	14.45	17	18.15	23.9	10.1	17.16	2.96	0.85	

HOx, patients with hyperoxaluria; noHOx, patients without hyperoxaluria; Ctrl Cr/Ox, control group for comparison of creatinine and urinary oxalate excretion to short bowel syndrome groups; Ctrl CysC control group two for comparison of eGFR to short bowel syndrome groups.

## Results

### Demographics and disease entities in hyperoxaluric patients

Fifty six patients were identified in the described period with diagnosis of short bowel syndrome. Out of these patients, analysis of urinary oxalate excretion was performed in 24 cases as two patients were anuric. In sum, a total of 26 patients were included into the study. Patient characteristics are depicted in Table 1, an overview of the most relevant parameters of each patient is shown in Tables 2, 3. Subjects with hyperoxaluria were 9 female (64%) and 5 male patients, subjects without hyperoxaluria were 3 female (25%) and 9 male patients. There was no statistically significant difference in gender distribution between the two groups, although the statistical significance level was just missed (*p* = 0.0618). There were no significant differences regarding BMI or age.

**Table 2:** Overview of the most important parameters of each patient in the group with hyperoxaluria.

Patient number	BMI (kg/m^2^)	Age at oxalate evaluation (months)	Etiologiy of SBS	Urinary oxalate (mmol/mol creatinine)	Creatinine (mg/dl)	eGFR (ml/min/1.73 m^2^)	Renal pathology	Remaining expected short bowel length (%)
1	14.6	15	NEC	138	0.21	186.91	NC	14
2	14.7	63	Volvulus	123	0.21	126.13	None	37
3	15.8	40	Intestinal atresia	115	0.22	132.51	None	32
4	14.7	107	NEC	na	6.53	5.26	dPC	26
5	20.3	154	Volvulus	283	0.31	133.80	NL	21
6	16.86	176	NEC	82	0.31	103.79	None	16
7	14.4	44	Volvulus	126	0.32	113.76	None	27
8	18.85	32	Volvulus, Gastroschisis	120	0.36	108.55	None	9
9	15.7	46	Volvulus, NEC	119	0.37	174.13	None	14
10	22.9	194	Volvulus, Gastroschisis	43.8	0.39	na	None	34
11	18.6	33	Volvulus	229	0.54	na	None	24
12	15.9	50	NEC	244	0.66	139.16	None	22
13	11.96	22	Volvulus	156	0.26	162.54	None	21
14	16.5	278	Intestinal scarring stenosis	na	18.5	2.2	dPC	X

NC, nephrocalcinosis; NL, nephrolithiasis; dPC, diffuse parenchymal calcifications.

**Table 3:** Overview of the most important parameters of each patient in the group without hyperoxaluria.

Patient number	BMI (kg/m^2^)	Age at oxalate evaluation (months)	Etiology of SBS	Urinary oxalate (mmol/mol creatinine)	Creatinine (mg/dl)	eGFR (ml/min/1.73 m^2^)	Renal pathology	Remaining expected short bowel length (%)
15	18.16	42	NEC	19	0.35	92.83	None	na
16	13.8	71	Intestinal atresia, Gastroschisis	49	0.26	113.85	None	17
17	23.9	179	NEC	27	0.77	na	None	11
18	18.18	7	NEC	140	0.34	104.51	None	44
19	16.2	126	Volvulus	15	0.34	158.53	None	26
20	17.81	27	Intestinal atresia	69	0.24	na	None	17
21	16.8	12	NEC	155	0.3	na	None	16
22	15.07	60	NEC	54	0.33	77.48	NC	5
23	17.2	117	Volvulus	29	0.42	102.83	None	11
24	13.9	70	NEC	12	0.42	110.73	None	na
25	14.24	72	Intestinal atresia	51	0.31	140.57	None	na
26	20.7	186	Meckel's diverticula	5.5	0.72	90.83	None	10

NC, nephrocalcinosis.

The etiologies for the development of SBS are shown in Table 4. There was a noticeable but statistically not significant clustering of the diagnosis volvulus in the group with hyperoxaluria (8/14 vs. 2/12). On the other hand, 80% (8/10) of patients with volvulus as the underlying disease developed hyperoxaluria (*p* = 0.023).

**Table 4:** Disease distribution between the two SBS groups.

Group	NEC	Gastroschisis	Volvulus	Jejunal or ileal atresia	Intestinal scarring stenosis	Meckel's diverticulum
HOx	5	2	8	1	1	0
nOHx	6	1	2	3	0	1

HOx, patients with hyperoxaluria; noHOx, patients without hyperoxaluria; NEC, necrotizing enterocolitis; SBS, short bowel syndrome.

Among these 56 patients, two suffered from terminal renal insufficiency, one of whom having concomitant intellectually disabled is now over 18 years of age. Each of these two patients had undergone small bowel resections in the past. Both patients are anuric, so urine examination was impossible. In both patients, renal organ transplantation was performed. Both lost their transplants only a few days after transplantation. In histological examination oxalate depositions could be detected. In addition, repeatedly very high plasma oxalate levels were measured (maximum 166 and 207 µmol/L, respectively) after organ failure, making the diagnosis enteric hyperoxaluria likely.

### Assessment of lithogenic parameters in SBS patients

**Figure 2:**
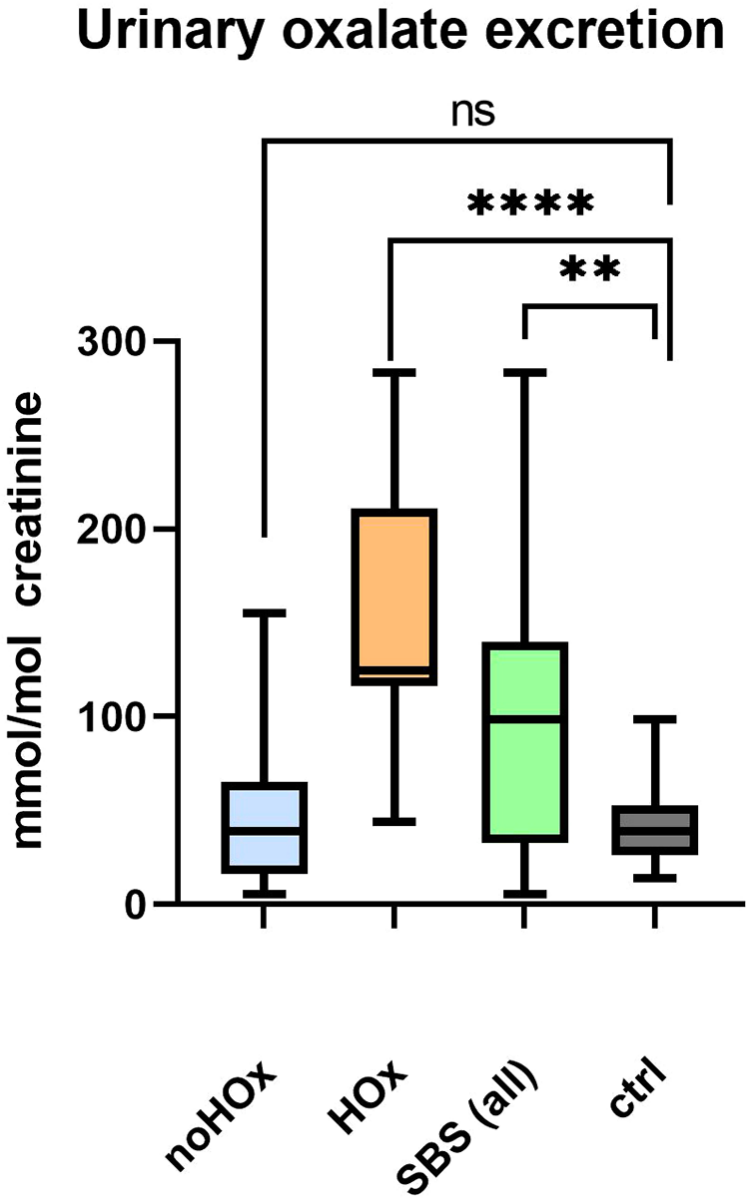
Urinary excretion of oxalate per creatinine between the different groups showing minimal to maximal values. Significantly higher oxalate excretion between whole short bowel patients and a control group can be demonstrated. No higher oxalate excretion was seen in the non-hyperoxaluric short bowel group and the control group, but a tendency towards a higher oxalate excretion is seen. **, *p* ≤ 0.01; ****, *p* ≤ 0.0001; ns, not significant; HOx, patients with hyperoxaluria; noHOx, patients without hyperoxaluria; SBS (all), all patients with short bowel syndrome.

Overall, 12 out of a total of 24 patients (50%, two patients were anuric therefore no urine collection was possible) had an elevated oxalate/creatinine ratio either in the past or currently at least once. One patient was already receiving dietary treatment for enteric hyperoxaluria. Therefore, data before the start of dietary therapy were taken into the assessment. In the hyperoxaluria group, a higher median oxalate excretion of 124.5 mmol/mol creatinine was measured compared to the group without hyperoxaluria (39 mmol/mol; *p* = 0.0018). In controls, the measured urine oxalate/creatinine ratio was 38.85 mmol/mol. We found no statistically significant difference of oxalate excretion in controls compared to short bowel patients without hyperoxaluria (*p* = 0.8363) but, as per definition expected, to hyperoxaluric SBS patients (*p* < 0.0001). When the excretion of oxalate of all patients with SBS was compared to controls, there was a significantly higher excretion of oxalate seen in the SBS group (98.5 mmol/mol, *p* = 0.0017). Data is shown in Table 5 and Figure 2. No statistical differences could be found with regard to the excretion of calcium or citrate between the two SBS groups.

**Table 5:** Laboratory parameters.

Parameter	Group	Number of values	Minimum	25% percentile	Median	75% percentile	Maximum	Range	Mean	Std. deviation	Std. error of mean	*p* value
Creatinine (mg/dl)	HOx	14	0.21	0.25	0.34	0.54	18.5	18.29	2.08	5.0	1.34	0.8693
	noHOx	12	0.24	0.3	0.34	0.42	0.77	0.53	0.4	0.17	0.05	
	Control group	50	0.25	0.31	0.38	0.45	0.86	0.61	0.4	0.12	0.017	0.5939 (to noHOx) 0.5705 (to HOx)
eGFR (ml/min/1.73 m^2^)	HOx	12	2.2	105	129.3	156.7	186.9	184.7	115.7	58.17	16.79	0.2773
	noHOx	9	77.49	91.83	104.5	127.2	158.5	81.04	110.2	25.26	8.42	
	Control group	28	87.29	108.3	119.2	142	162.6	75.31	123.9	21.08	3.98	0.1164 (to noHOx) 0.8729 (to HOx)
Urinary oxalate (mmol/mol creatinine)	HOx	12	43.8	116	124.5	211	283	239.2	148.3	69.55	20.08	**0** **.** **0018**
	noHOx	12	5.5	16	39	65.25	155	149.5	52.13	48.65	14.04	
	SBS (all)	24	5.5	32.7	98.5	139.5	283	277.5	100.2	76.54	15.62	**0** **.** **0017**
	Control group	50	14.1	26.3	38.85	52.55	98.7	84.6	40.73	17.77	2.51	
Urinary citrate (mmol/mol creatinine)	HOx	10	11.9	105.9	235.5	505.8	842	830.1	318.2	277.5	87.75	0.7361
	noHOx	12	0.79	32.25	221	612.8	1,521	1,520	376.4	466.7	134.7	
Urinary calcium (mol/mol creatinine)	HOx	12	0	0.34	0.62	1.07	3.67	3.67	0.952	1.04	0.3	0.8874
	noHOx	12	0.1	0.27	0.58	1.64	2.37	2.27	0.9	0.76	0.22	

HOx, patients with hyperoxaluria; noHOx, patients without hyperoxaluria; SBS (all), all patients with short bowel syndrome.

Bold values highlight statistical significance (*p* < 0.05).

### Assessment of renal function and renal pathology in SBS patients

Looking at the creatinine concentrations as a marker of renal function, no statistical difference was seen between the two SBS groups. Cystatin C concentrations could be determined in 21 out of 26 patients. We found a higher eGFR of 129.3 ml/min/1.73 m^2^ in the group with hyperoxaluria compared to 104.5 ml/min/1.73 m^2^ in those without but statistical significance was not reached (*p* = 0.5705, see Table 5). There was no significant difference between the eGFR or creatinine of the healthy control group compared to any of the two SBS groups.

The incidence of nephrocalcinosis or nephrolithiasis was 4/14 (28.6%) in the hyperoxaluric group. One of these patients had pathological renal ultrasound findings (1/14, 21%) characterized by hyperechoic cortical halo compatible with a beginning cortical nephrocalcinosis in both sides (ultrasound of patient 1 is shown in Figure 1), two patients (2/14, 14%) had diffuse parenchymal calcifications in both sides. One patient developed nephrolithiasis (1/14; 7%). In the non-hyperoxaluric group, only one patient was diagnosed with echogenic cortical halo being consistent with a beginning mild cortical nephrocalcinosis (1/12, 8.3%). However, there was no statistically significant difference between the two SBS groups.

### Evaluation of the impact of the residual small and large intestinal length on hyperoxaluria

**Figure 3:**
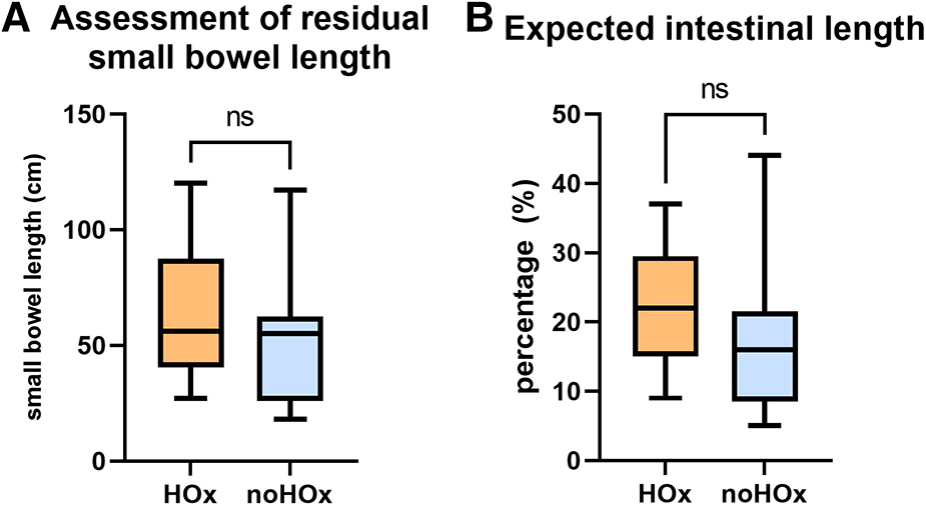
Residual small bowel length (**A**) and percentage of expected small bowel length (**B**) between the group with and without hyperoxaluria. No statistical significance could be seen between the two groups. ns, not significant; HOx, patients with hyperoxaluria; noHOx, patients without hyperoxaluria.

In the group with hyperoxaluria median small intestinal length averaged 56 cm compared to 55 cm in the group without hyperoxaluria. The expected intestinal length as described by Struijs' criteria (19) was 22% in the group with hyperoxaluria and 16% in the group without hyperoxaluria (depicted in Figures 3A,B and Table 6). A statistical correlation between urinary oxalate excretion and anatomical length of the remaining small intestine could not be observed, which was also the case for large intestinal length and urinary oxalate excretion (see Table 6). Bowel continuity was present in all (*n* = 14) of the patients with hyperoxaluria, only 1/12 (8%) in the group without hyperoxaluria did not show bowel continuity. A statistically significant difference could not be calculated. There were no differences in stool frequency between the groups.

**Table 6:** Clinical variables.

Parameter	Group	Number of values	Minimum	25% percentile	Median	75% percentile	Maximum	Range	Mean	Std. deviation	Std. error of mean	*p* value
Stool frequency (x/day)	HOx	14	1	1	2.25	3.25	7	6	2.61	1.64	0.43	0.7651
	noHOx	10	1	1	2	4	4	3	2.3	1.27	0.4	
Small intestinal length (cm)	HOx	13	27	10.5	56	87.5	120	93	62.62	28.48	7.9	0.5228
	noHOx	9	18	26	55	62.5	117	99	53.22	29.64	9.88	
Residual expected small intestinal length (%)	HOx	13	9	15	22	29.5	37	28	22.85	8.37	2.32	0.1216
	noHOx	9	5	8.5	16	21.5	44	39	17	11.94	3.98	
Large intestinal length	HOx	13	9	32.5	45	64.5	119	97	51.92	27.85	7.72	0.8321
	noHOx	7	5	27	53	134	139	126	65.57	50.95	19.26	

HOx, patients with hyperoxaluria; noHOx, patients without hyperoxaluria.

### Evaluation of the impact of enteral nutrition on oxalate excretion

While in the group with hyperoxaluria 11 out of 14 patients (78.5%) were completely enteral fed and did not receive any type of parenteral nutrition, this was the case in only 7 out of 12 (58%) of the patients without hyperoxaluria. Statistical significance could not be met (*p* = 0.4). Therefore, no statistically significant correlation between type of nutrition and a higher renal oxalate excretion could be seen.

## Discussion

Patients with short bowel syndrome have heterogenous entities, treatment is often very complex and demanding. The main aspect in medical care for these patients, especially in the pediatric setting, is nutrient supply. Chronic intestinal failure describes the inability of the gut to maintain adequate nutrient and fluid intake for homeostasis in adults or for adequate growth in children without parenteral supplementation (29) with the need of parenteral nutrition for at least 60 days within a consecutive 74 day interval according to the American Society for Parenteral and Enteral Nutrition in pediatrics (30).

In pediatric patients, the most common cause of intestinal failure is anatomical SBS, which accounts for about 85% (31). Pediatric patients with SBS face a variety of clinical problems reaching from severe infections, IFALD and malnutrition to metabolic disturbances thus having an increased need for adequate and continuous medical care. Enteric hyperoxaluria as a complication in patients with SBS is rarely addressed so far. It can be associated with nephrocalcinosis, nephrolithiasis or even renal insufficiency.

**Figure 4:**
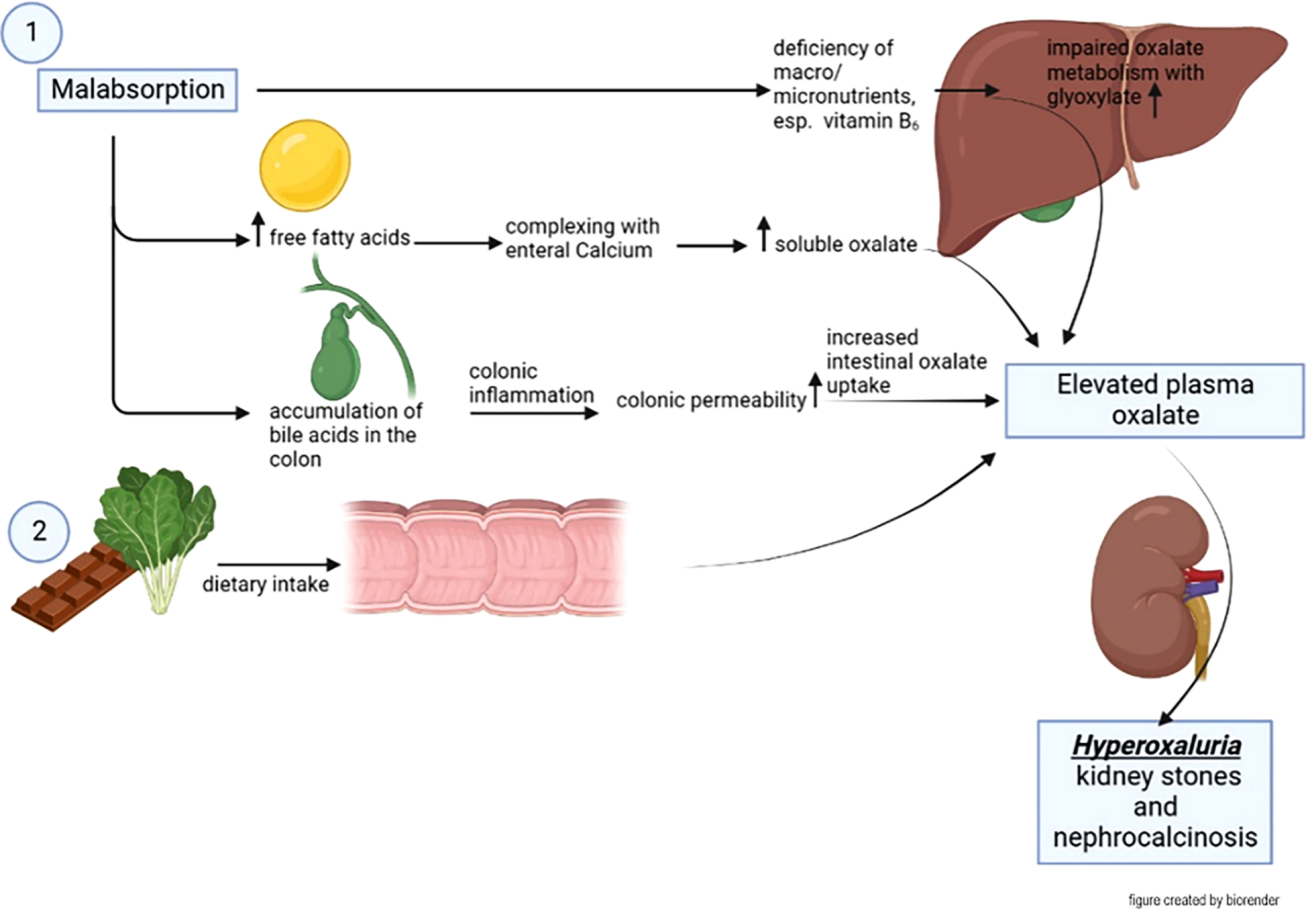
Pathomechanism of enteric hyperoxaluria. Malabsorption results in an increase of free fatty acids in the small intestine. These form complexes with calcium, which among others is bound to oxalate. Free oxalate in turn is much easier absorbed by the intestine. On the other hand, free bile acids lead to colonic inflammation which lead to a higher uptake of oxalate. These factors taken together have the consequence of a higher uptake of oxalate especially in the colon which can lead to nephrolithiasis or nephrocalcinosis [adapted by Nazzal et al. (32)].

Genetically determined forms of hyperoxaluria (primary hyperoxaluria) need to be distinguished from secondary forms of hyperoxaluria, which can be caused by excessive enteral oxalate intake (or its precursors like vitamin c) or increased absorption [see Figure 4, adapted by Nazzal et al. (32)], the latter being called enteric hyperoxaluria. Enteric hyperoxaluria has been observed in patients with Crohn's disease, cystic fibrosis, pancreatic insufficiency or after Roux-en-Y gastric bypass surgery (RYGB) (32–36).

Usually, oxalate is bound to calcium in the intestine, which reduces its absorption capacity. In an acidic environment, this bond is loosened leading to an increased mucosal uptake of free oxalate (37). Another possibility to break this bond is a displacement of calcium from oxalate by free fatty acids, whereby a calcium soap is formed (38, 39). Under physiological conditions and normal anatomy, especially bile acids (and thus also long-chain fatty acids) are reabsorbed in the terminal ileum, so that only a small amount of the excreted bile acids pass into the colon. In case of an absence of the terminal ileum, unphysiologically large amounts of bile acids and free fatty acids entering the colon cause an inflammatory reaction leading to mucosal barrier dysfunction which causes an increased oxalate absorption in the colon (39–41). Thus, the mucosal barrier dysfunction in combination with increased free oxalate in the colon leads to a disproportionate colonic absorption of oxalate.

Here, we observed a high prevalence of secondary hyperoxaluria in nearly 55% of the patients from our cohort. Compared to controls, SBS patients showed a significantly higher urinary oxalate excretion. In line with this are recent data from Guz Mark et al. who investigated the renal excretion of oxalate in patients with chronic intestinal failure, seven of them with anatomical SBS (2). Of our 26 patients 14 showed hyperoxaluria, 4 of them developed nephrocalcinosis or nephrolithiasis. In a recent study by Roberts and coworkers, even 63% patients were reported with hyperoxaluria (3) and of these, 5 (38.5%) had alterations in the renal ultrasound although not fulfilling the criteria for nephrocalcinosis in all points.

Regarding nephrocalcinosis Billing describes an incidence of 18% in his cohort of 50 children on long-term parenteral nutrition (42). Kosar et al. also studied sonographic renal pathologies in 56 children with chronic intestinal failure (76% of those with short bowel) (17). These were defined as increase in echogenicity, nephrolithiasis or nephrocalcinosis. 43% showed such renal abnormalities, although details on how this number was composed of were not described. In their study, oxalate excretion in the group with sonographic abnormalities did not differ from that without renal abnormalities.

In our study and comparable with the study from Billing et al. (42), nephrocalcinosis or nephrolithiasis was found in 19% (5 out of 26) of all patients examined, with this being the case in 28.6% (4 out of 14) of patients with hyperoxaluria. A statistically significant difference between the groups with and without hyperoxaluria could not be provided. An explanation for this observation might be the limited number of patients. In addition, the development of nephrolithiasis and nephrocalcinosis is multifactorial. Known causes include hypercalciuria, hyperoxaluria, hypocitraturia and hypomagnesuria, as well as dehydration and various other metabolic changes (43). It is likely that nephrolithiasis also develops independently of hyperoxaluria in short bowel patients and that this is not the only causative factor, as short bowel patients are a risk group for all the risk factors mentioned. Therefore, it is possible that hyperoxaluria as an isolated risk factor for nephrocalcinosis or nephrolithiasis only becomes statistically significant in a larger cohort.

Surprisingly, 80% of the patients with volvulus developed enteric hyperoxaluria, which has not been described previously. Several aspects like a shorter remaining intestinal length or a higher rate of ileocecal resection in patients with volvulus could be responsible for this observation. In our cohort we could not see such a correlation (data not shown).

Our cohort provides additional information on renal function in the longitudinal course. In this regard, we did not observe an increase in serum creatinine concentration or a reduction in eGFR calculated by Grubb's formula over time compared to controls. Furthermore, there were no differences seen between the group with and without hyperoxaluria. Few studies reported a reduced eGFR in pediatric patients on parenteral nutrition therapy (44). In the study by Ylinen et al. including 70 patients with pediatric intestinal failure on parenteral nutrition for at least 1 month, a GFR reduction in 29% of the patients studied was reported over an observation period of 3.2 years on average (45), whereby a statistical significance of the creatinine compared to the group without creatinine increase could not be established. Billing, on the other hand, found chronic renal failure (determined on the basis of 24-h creatinine clearance) in 30% of pediatric patients with chronic intestinal failure (42). In addition, he was able to show that proteinuria, an early marker of renal function impairment, was detectable in up to 76% of patients. It is worthful to mention that, at least in parts, overflow proteinuria plays a role in patients with (partial) parenteral nutrition. Therefore, proteinuria in patients fed with parenteral nutrition might not necessarily be a marker for renal damage as it is in patients with diabetes mellitus or without parenteral nutrition.

The length of the remaining small intestine and colon is crucial for the clinical course and the extent of (mal)absorption of a patient with SBS. Based on intestinal growth, Struijs et al. established standard curves for expected small intestinal length in the first 5 years of life (19). In the present study, intestinal length was assessed by intraoperative measurements and by radiological assessment on the one hand and by radiological assessment on the other. A positive correlation between small bowel length (absolute and relative according to Struijs) and oxalate excretion could not be determined, as in both groups the intestinal length was comparable. Hence, no statistical difference could be demonstrated. This is in line with the data from Kosar and Rudzinski as they also could not find a correlation between intestinal length and the occurrence of oxalate excretion or sonographic changes/nephrolithiasis (16, 17). Concerning intestinal length, it should be added that the site of resection also is a relevant aspect.

It is worthful to mention that in the group without hyperoxaluria one patient (#18) had undergone a moderate small bowel resection with a remaining short bowel length of 44%, representing the patient with the longest remaining small intestine within the whole cohort. Additionally, in this patient the criteria of SBS according to Muto et al. (18) have barely been fulfilled as parenteral nutrition was applied just a few days longer than 2 months after short bowel resection was performed. We would like to mention that omission of this patient from analysis would have led to a statistically relevant correlation between remaining expected short bowel length and hyperoxaluria with hyperoxaluric patients displaying a slightly longer remaining bowel length (median 13.5%, interquartile range for patients without hyperoxaluria = 21%, *p* = 0.028). One possible explanation for this observation could be that a certain intestinal contact time requires and affects the quantity of oxalate to be intestinally absorbed. If nutrition including oxalate content passes too fast, less absorption happens. Therefore, SBS patients with a very short remaining small bowel (e.g., <15%) might have a lower risk for development of hyperoxaluria than patients with a remaining short bowel length of 25%–30%.

Results regarding enterocolic continuity and hyperoxaluria are somewhat more divergent. While Nightingale and Yang found that adult patients with intestinal continuity had an increased risk of nephrolithiasis (15, 41), this was not the case in studies by Rudzinski and Kosar. On the other hand, several publications have shown the pathophysiological importance of the colon in enteric hyperoxaluria (32, 38, 46–48). In our study, no correlation between intestinal continuity and development of enteric hyperoxaluria could be seen. Further studies with more patients are necessary to clarify if absence of certain intestinal sections, the remaining bowel length and/or intestinal continuity represent risk factors for the development of enteric hyperoxaluria in SBS patients, as the generally small sample size might be responsible for inconclusive study results.

Our study contains a number of limitations, e.g., the monocentric and retrospective study design and the different methods of assessing the residual intestinal small bowel length. As mentioned above, the gold standard for establishment of the diagnosis enteric hyperoxaluria is a 24-h urine collection accompanied by an oral load of oxalate. This was not possible in the most cases of our patients as the majority of these patients were urinary incontinent. Additionally, only existing data could be analysed and 24-h urine collection was not performed in routine clinical care therefore making a spot urine the most applicable sampling method. In addition, the evaluation of possible microbiota alterations in our cohort would have been of interest, e.g., colonisation status with Oxalobacter formigenes. However, this will be the aim in our future studies.

In summary, our study found no correlation between enteric hyperoxaluria and intestinal length, renal function or other laboratory parameters. The proportion of SBS patients having an increased renal oxalate excretion was about 55% in our cohort and thus unexpectedly high. It was striking that hyperoxaluria occurs in 80% of patients with volvulus resulting in SBS. The reason for this finding is not clear. The intestinal length in patients with volvulus was not shorter than in the rest of the SBS-group. Maybe the affected anatomical part impacts mucosal-intestinal resorptive functions in patients with volvulus that might differ from the others. Larger studies from national or international SBS-registries are warranted to clarify the assumed correlation of SBS etiology, remaining small bowel and colon length with hyperoxaluria and nephrocalcinosis or nephrolithiasis. Furthermore, these studies could elucidate a correlation between hyperoxaluria and the progression of renal function impairment in pediatric patients, since oxalate-lowering therapy strategies such as the administration of Oxalobacter formigenes or oral enzyme therapy (e.g., oxalate decarboxylase) could possibly be advantageous for this patient group if dietary measures are not sufficient.

## Data Availability

The original contributions presented in the study are included in the article, further inquiries can be directed to the corresponding authors.
